# Reviewing Evidence and Patient Outcomes of Cheilectomy for Hallux Rigidus: A Systematic Review and Meta-Analysis

**DOI:** 10.3390/jcm13237299

**Published:** 2024-11-30

**Authors:** Alberto Arceri, Gianmarco Di Paola, Antonio Mazzotti, Simone Ottavio Zielli, Elena Artioli, Laura Langone, Federico Sgubbi, Cesare Faldini

**Affiliations:** 1 1st Orthopaedics and Traumatologic Clinic, IRCCS Istituto Ortopedico Rizzoli, 40136 Bologna, Italy; alberto.arceri@ior.it (A.A.); gianmarco.dipaola@ior.it (G.D.P.); simoneottavio.zielli@ior.it (S.O.Z.); elena.artioli@ior.it (E.A.); laura.langone@ior.it (L.L.); federico.sgubbi@ior.it (F.S.); cesare.faldini@ior.it (C.F.); 2 Department of Biomedical and Neuromotor Sciences (DIBINEM), Alma Mater Studiorum University of Bologna, 40123 Bologna, Italy

**Keywords:** hallux rigidus, cheilectomy, minimally invasive, ROM, pain

## Abstract

**Background:** Cheilectomy is a joint-sparing surgery for the treatment of moderate stages of Hallux Rigidus (HR). The purpose of this systematic review was to assess the clinical outcomes, range of motion (ROM), complications, and revision rates associated with cheilectomy. **Methods:** A literature search of the PubMed, Scopus, and Cochrane databases was performed. PRISMA guidelines were used. Risk of bias was assessed through the Newcastle–Ottawa Scale. Meta-analysis of the clinical outcomes scores was performed. **Results:** The initial search identified 317 articles, with 16 included. Cheilectomy improved ROM by 51.15% (41.23° to 62.32°), with greater gains in traditional (67.72%) vs. minimally invasive (48.74%) techniques. VAS decreased by 72.61%, more in traditional (79.35%) than minimally invasive (64.97%). AOFAS improved by 33.99%, from 61.83 to 82.85. Complications occurred in 11% (11.68% traditional, 9.73% minimally invasive), with residual pain (7.46%) more common in traditional and nerve injury (3.78%) in minimally invasive procedures. Revision rates were 7.4% overall (6.1% traditional, 8.8% minimally invasive). **Conclusions:** This procedure showed satisfactory results regardless of whether the traditional or minimally invasive technique is used. Current evidence does not allow for a definitive indication, but careful patient selection is advisable, particularly for mild to moderate cases.

## 1. Introduction

The term Hallux Rigidus (HR) was introduced by Cotterill in 1888 to describe a pathological condition characterized by pain and functional limitation of the first metatarso-phalangeal joint (FMTPJ) [1]. HR is the second most common pathology of the hallux after valgus deformity, accounting for 9.3% of patients visiting a foot and ankle surgeon [2,3]. This condition predominantly affects women over 50 years old [4].

Treatment is guided by the disease stage, utilizing different scoring systems. The parameters evaluated for HR staging include clinical and radiographic findings, considering pain levels, FMTPJ range of motion (ROM), joint space narrowing, osteophyte formation, and morphological alterations of the affected bones [5,6]. The most widely used system to classify HR grade, proposed by Hattrup and Johnson in 1988 and later modified by Coughlin and Shurnas, consists of four degrees based on dorsiflexion stiffness and pain [5,6] (Table 1).

Conservative treatment represents the first option in the early stages of HR and involves orthoses, physical therapy, and drugs such as NSAIDs and steroids [7,8]. Surgical treatment is indicated in cases where conservative measures fail. Several procedures, including joint-preserving and -sacrificing options, such as cheilectomy, resection or interpositional arthroplasties, FMTPJ prostheses and arthrodesis, have been described [9,10]. The main goal of treatment is to preserve motion and relieve pain, except in cases of end-stage disease where FMTPJ arthrodesis is considered the gold standard. Cheilectomy, first described by DuVries in 1959 [11], currently represents the most popular joint-sparing surgery for treating moderate stages of HR [10,12,13]. This procedure involves the excision of osteophytes from the metatarsal bone and phalangeal base, as well as removal of up to 30% of the dorsal head metatarsal joint surface, which limits FMTPJ dorsiflexion, causing stiffness and pain. The literature reports traditional open cheilectomy techniques involving either medial or dorsal incisions [10], as well as more recent minimally invasive approaches that rely on small, selective incisions through which a burr is introduced to smooth the bony surfaces [13]. A cut-off of 30% has been described in the literature to distinguish cheilectomy from the Valenti procedure [14,15,16,17].

The most recent systematic reviews date back to 2010 [18]. Given the passage of over a decade, it is important to reassess the outcomes and complications reported in the recent literature to determine whether significant changes or improvements have occurred in the field. Therefore, the purpose of this systematic review was to assess the clinical outcomes, ROM, complications, and revision rates associated with cheilectomy.

## 2. Materials and Methods

### 2.1. Search Strategy and Selection Criteria

This systematic review adhered to the PRISMA (Preferred Reporting Items for Systematic Reviews and Meta-Analyses) guidelines [19], although a formal protocol for the systematic review was not registered. This choice was made to improve the authors’ flexibility to adapt the research in response to emerging information or necessary changes during the review process, thereby enhancing the relevance and timeliness of the results, given the challenging data interpretation.

A comprehensive search was conducted across PubMed, Scopus, and the Cochrane Library databases using the same inclusive text-based query for all: “hallux rigidus OR hallux limitus OR first MTP arthritis OR big toe arthritis AND cheilectomy OR cheilotomy OR exostectomy”, where all upper keywords represent the Boolean operators used. Date restriction has been applied: only last-ten-year articles published from January 2013 to June 2024 were selected. Additionally, a thorough search was performed within the reference lists of all included articles to identify pertinent studies. Two authors (G.D.P. and F.S.) independently conducted the literature search.

Studies were eligible for inclusion if they were consistent with the reported clinical or radiological outcomes of cheilectomy for HR. Exclusion criteria were studies with missing data on outcomes; studies reporting additional surgical procedures or more than 30% metatarsal head resection; studies with fewer than 10 participants; biomechanical or cadaveric studies; reviews, meta-analyses, case reports and letters to the editor; non-English articles were also excluded.

### 2.2. Study Selection and Data Collection

Following the removal of duplicate entries, two authors (G.D.P. and F.S.) independently reviewed the titles and abstracts of the remaining papers to determine their eligibility based on inclusion and exclusion criteria. Full-text versions of the relevant articles were then obtained for further assessment. Any disagreements regarding article inclusion were resolved by consulting the senior author (C.F.).

Study characteristics retrieved from the studies encompassed author and publication year, study design, and the level of evidence (LOE). Information extracted from the included papers was organized based on the PICO question (participants, intervention, comparisons, and outcomes):

- Participants: number of patients (and toes) undergoing cheilectomy, patient’s sex, mean age at surgery and follow-up;
- Intervention: type of surgical procedure (traditional cheilectomy, mini-invasive cheilectomy);
- Comparisons: comparison of clinical scores and FMTPJ ROM between cheilectomy approaches;
- Outcomes: clinical outcomes, ROM, complications, and revision rate relative to different cheilectomy approaches.

Data collection and organization were facilitated using Microsoft Excel 360 (Microsoft Corporation, Redmond, WA, USA) for Windows 11.

### 2.3. Quality Assessment

The Newcastle-Ottawa Scale was adopted to assess the quality and potential bias of the included studies [20]. Two reviewers (G.D.P. and F.S.) independently conducted the quality assessment (risk of bias) for the studies included. This scale consists of eight items divided into three categories. The quality score can range from 0 to 9 stars. Studies with a score ≥5 are classified at low risk of bias, while those scoring <5 points are considered at high risk of bias.

### 2.4. Statistical Analysis

Descriptive analysis was performed. Continuous variables were presented as mean values and standard deviation or range, while categorical variables were expressed as frequencies and/or percentages. When appropriate, a chi-square test was performed to determine differences in categorical variables. A weighted average of the continuous variables for different techniques was calculated, followed by a descriptive analysis.

Studies sharing the same clinical outcomes with available mean and standard deviation, or range of the pre- and post-operative score values, were considered eligible for meta-analysis. The analysis was performed if applicable to at least four studies, and only studies scoring ≥5 on the Newcastle-Ottawa Scale were included, as scores below 5 were classified as high risk. This selection criterion minimized the inclusion of high-risk studies, ensuring comparability and reducing bias in our results.

All the statistical analysis was carried out using the software Jamovi project (2022) version 2.3, retrieved from https://www.jamovi.org.

## 3. Results

**Figure 1:**
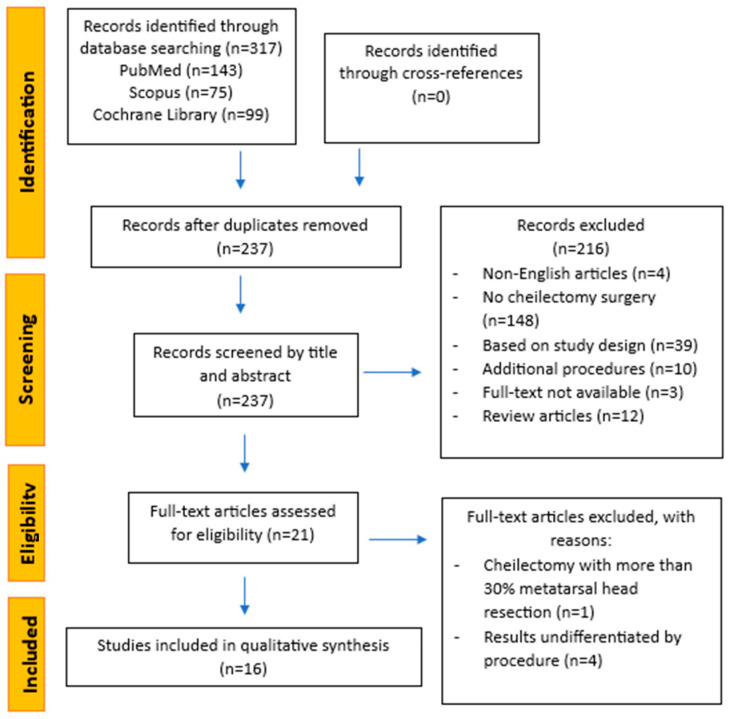
Flowchart of review process by PRISMA.

Database research identified 317 potentially eligible papers. After manual duplicate removal, 237 remaining studies were reviewed by title and abstract to select those eligible for inclusion in the review, resulting in the selection of 21 full-text articles for further evaluation of eligibility. No records were identified through cross-referencing. At the end, 16 articles fulfilled the inclusion criteria and were included in the present systematic review (Figure 1).

### 3.1. Quality Assessment

The assessment of bias risk in the included studies, as measured by the Newcastle–Ottawa Scale, is reported in Table 2. Only three studies were evaluated as having a high risk of bias; the remaining 13 studies achieved a score of 5 or more stars, classifying them as low risk of bias.

Of the included studies, nine (56.25%) were retrospective case series classified as LOE IV according to the Oxford Level of Evidence scale, five (31.25%) were retrospective comparative studies classified as LOE III, and two (12.5%) were prospective comparative studies classified as LOE II (Table 3). The selected articles were published from 2014 to 2024 (Table 2).

### 3.2. Population

The total amount of patients undergoing cheilectomy across included studies was 1133. In 46 cases, the procedure was bilateral, resulting in a total of 1179 treated halluces. Females were the predominant gender: 735 female (66.4%) vs. 372 male (33.6%). Only one study did not provide information about gender distribution [31]. The mean age of patients at surgery was 54.1 years (range 16–79). The mean clinical and radiological follow-up was 3.5 years. The demographics data are summarized in Table 3.

### 3.3. Clinical Outcome Scores

All clinical outcomes were reported in Table 4. Only the 27 halluces undergoing traditional cheilectomy in the Galli, S.H. et al. study [25] were included in the analysis of outcomes.

Pain symptoms were assessed through the VAS in nine studies [22,25,26,27,28,30,34,35,36]. Of these, eight studies [22,25,26,27,30,34,35,36], encompassing 395 halluces, reported both pre-operative and post-operative mean VAS scores, while one study [28] provided only the post-operative value. The weighted mean VAS was 6.61 pre-operatively and 1.82 postoperatively, with a 72.61% reduction in pain. When differentiating between the two surgical techniques, the traditional cheilectomy (246 halluces) showed a pre-operative VAS of 6.20 and a post-operative VAS of 1.29, indicating a 79.35% pain reduction [22,25,30,34,35]. For the minimally invasive cheilectomy (149 halluces), the pre-operative VAS was 7.28, and the post-operative VAS was 2.55, representing a 64.97% improvement in pain [26,27,28,36] (Table 5).

Six papers [22,25,29,30,33,35] employed the American Orthopaedic Foot and Ankle Society Score (AOFAS) to assess clinical outcomes. A weighted mean AOFAS score, including 155 halluces, improved by 33.99% from 61.83 to 82.85 after cheilectomy. The other studies used various scores to assess clinical outcomes; these findings have been summarized in Table 4. Due to the wide variability in the clinical outcome measures used, it was not possible to investigate differences between surgical techniques.

The total ROM of the FMTPJ was measured in six studies [22,26,27,29,33,35]; however, five studies [22,26,27,29,33], including 151 halluces, reported both pre- and post-operative mean value. The weighted mean of total ROM was 41.23° pre-operatively and 62.32° after surgery, with a 51.15% improvement in motility (Table 4). The total ROM of patients undergoing traditional cheilectomy improved by 67.72% from 29.31° to 49.16° [22,29,35], while patients treated with a mini-invasive procedure started at 47.68° and increased to 70.92° [26,27,33], with a 48.74% improvement in joint mobility (Table 5).

Seven studies reported dorsiflexion measurement [26,27,28,29,33,35,36]. One study reported only the post-operative value [35]. The total weighted mean pre-operative dorsiflexion value improved from 25.44° to 59.88° [26,27,28,29,33,36] (Table 4). Only one paper concerning traditional cheilectomy reported both pre- and post-operative FMTPJ dorsiflexion, which started at 24° and increased to 38° after the procedure [29] (Table 5). Five papers on minimally invasive cheilectomy reported pre- and post-operative dorsiflexion values: the weighted average increased from 25.63° to 64.48° [26,27,28,33,36] (Table 5).

Five studies reported plantarflexion measurements [26,27,29,33,35]. The plantarflexion was 11.65° preoperatively and 15.51° post-operatively [26,27,29,33,35] (Table 4). Differentiating between surgical procedures, plantarflexion increased from 16.9° to 18.86° in patients treated with open surgery [29,35], and from 9.99° to 14.07° in patients undergoing mini-invasive techniques [26,27,33] (Table 5).

The ROM values reported by one study [25] are based on a different measurement system than those used in the others, making direct comparisons impossible.

### 3.4. Complications and Revision Rate

Complications were assessed in 11 out of 16 studies [21,22,25,26,27,28,29,30,32,34,36] (Table 6). The overall prevalence of post-cheilectomy complications, regardless of the surgical technique used, was 11%. Persistent pain was the most frequently observed complication, reported by 7.46% of patients [25,26,28,32,34]. Transient nerve injury exhibiting hypoesthesia, paresthesia or hallux numbness, was observed in 1.86% of patients [22,28,36].

Traditional cheilectomy demonstrated a complication prevalence of 11.68%, with postoperative pain being the most reported complication, occurring in 9.97% of cases [21,22,25,29,30,32,34] (Table 6). Minimally invasive cheilectomy reported complications in 9.73% of cases, with nerve injury being the most frequent complication, occurring in 3.78% of cases [26,27,28,36] (Table 6).

Other complications, such as delayed wound healing, infection, and tendon injuries, are less common (Table 6) and do not appear to be associated with any specific surgical technique.

Revision rate was evaluated in 13 out of 16 studies [21,22,24,25,26,27,28,29,30,31,32,33,34] involving 934 halluces. In 7.4% of cases, further surgical procedures were required for ongoing pain, worsening functional limitation, infection, or other complications. Reoperation was needed in 6.1% of patients undergoing traditional cheilectomy [21,24,25,29,30,31,32,34] and 8.8% of patients treated with mini-invasive surgery [26,27,28,33,36]. The most frequent revision procedure performed was the FMTP joint fusion, adopted in 60.9% of cases.

### 3.5. Statistical Analysis

In the meta-analysis, studies that did not provide a complete set of pre- and post-operative values or failed to report the standard deviation or other parameters necessary for its calculation were excluded.

The potential impact of high-risk studies was addressed by including only studies with a Newcastle–Ottawa Scale score ≥ 5. As studies varied by only a single Newcastle–Ottawa Scale point, any potential influence on the overall findings is statistically negligible.

The analysis was carried out using the standardized mean difference as the outcome measure. A random-effects model was fitted to the data. The amount of heterogeneity (i.e., tau^2^) was estimated using the restricted maximum-likelihood estimator [38]. In addition to the estimate of tau^2^, the Q-test for heterogeneity [39] and the I^2^ statistic are reported. In case any amount of heterogeneity is detected (i.e., tau^2^ > 0, regardless of the results of the Q-test), a prediction interval for the true outcomes is also provided. Studentized residuals and Cook’s distances are used to examine whether studies may be outliers and/or influential in the context of the model. Studies with a studentized residual larger than the 100 × (1 − 0.05/(2 × k))th percentile of a standard normal distribution are considered potential outliers (i.e., using a Bonferroni correction with two-sided alpha = 0.05 for k studies included in the meta-analysis). Studies with a Cook’s distance larger than the median plus six times the interquartile range of the Cook’s distances are considered to be influential. The rank correlation test and the regression test, using the standard error of the observed outcomes as predictor, are used to check for funnel plot asymmetry.

**Figure 2:**
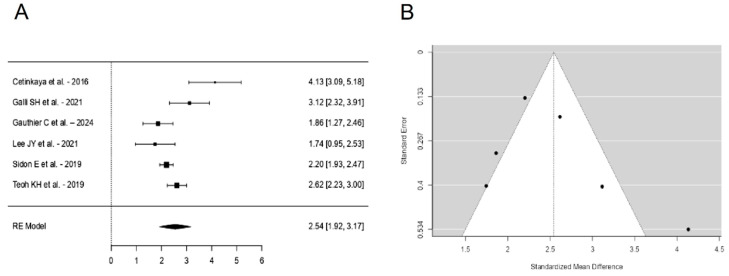
Forest plot (**A**) and funnel plot (**B**) of the VAS outcomes meta-analysis performed on six studies [22,25,26,30,34,36].

A total of k = 6 studies were included in the analysis. The observed standardized mean differences ranged from 1.7444 to 4.1342, with the majority of estimates being positive (100%). The estimated average standardized mean difference based on the random-effects model was \hat{\mu} = 2.5438 (95% CI: 1.9177 to 3.1698). Therefore, the average outcome differed significantly from zero (z = 7.9640, *p* < 0.0001). According to the Q-test, the true outcomes appear to be heterogeneous (Q(5) = 22.5982, *p* = 0.0004, tau^2^ = 0.4976, I^2^ = 87.5804%). A 95% prediction interval for the true outcomes is given by 1.0260 to 4.0615. Hence, even though there may be some heterogeneity, the true outcomes of the studies are generally in the same direction as the estimated average outcome. An examination of the studentized residuals revealed that one study [22] had a value larger than ±2.6383 and may be a potential outlier in the context of this model. According to the Cook’s distances, none of the studies could be considered to be overly influential. Neither the rank correlation nor the regression test indicated any funnel plot asymmetry (*p* = 0.1361 and *p* = 0.1949, respectively) (Figure 2).

**Figure 3:**
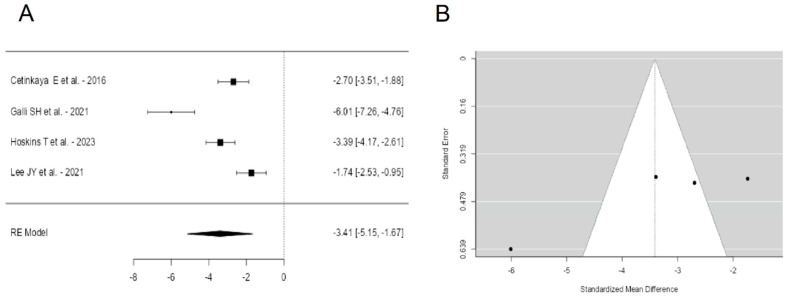
Forest plot (**A**) and funnel plot (**B**) of the AOFAS outcomes meta-analysis performed on four studies [22,25,29,30].

A total of k = 4 studies were included in the analysis. The observed standardized mean differences ranged from −6.0073 to −1.7375, with the majority of estimates being negative (100%). The estimated average standardized mean difference based on the random-effects model was \hat{\mu} = −3.4106 (95% CI: −5.1500 to −1.6712). Therefore, the average outcome differed significantly from zero (z = −3.8432, *p* = 0.0001). According to the Q-test, the true outcomes appear to be heterogeneous (Q(3) = 33.4954, *p* < 0.0001, tau^2^ = 2.9284, I^2^ = 93.6341%). A 95% prediction interval for the true outcomes is given by −7.1888 to 0.3676. Hence, although the average outcome is estimated to be negative, in some studies the true outcome may in fact be positive. An examination of the studentized residuals revealed that one study [25] had a value larger than ±2.4977 and may be a potential outlier in the context of this model. According to the Cook’s distances, none of the studies could be considered to be overly influential. The regression test indicated funnel plot asymmetry (*p* = 0.0029) but not the rank correlation test (*p* = 0.7500) (Figure 3).

## 4. Discussion

The present systematic review and meta-analysis of the current literature on cheilectomy applied to HR assessed clinical outcomes, ROM, complications, and revisions rate after surgery.

Studies published in the last ten years have confirmed the epidemiological trend of HR, which predominantly affects the female population over 50 years old [4,18,40].

Many authors consider cheilectomy a viable option for treating cases of mild to moderate HR, characterized by the presence of osteophytes limiting the FMTPJ ROM, with a mild to moderate reduction in joint space and an absence of major deformities [10,41,42,43]. These categories align with grades I and II of Coughlin and Shurnas and correspond to grade I, as well as milder cases of grade II, in the Hattrup and Johnson classification system. Many of the studies included in this systematic review reported the grading of hallux rigidus. Despite various classification systems being used, the authors generally adhered to the same criterion: the cases included were predominantly classified as mild to moderate [21,23,24,28,30,32,33,34,36]. In 10 studies [21,22,23,28,30,31,32,33,34,36], some patients (*n* = 243) were classified as having a grade higher than 2. However, no distinction was made in the clinical and functional outcomes based on the grading. Consequently, it was not possible to determine whether worse outcomes correlate with higher-grade cases. Therefore, the current available data do not allow assessment of the appropriateness of cheilectomy as a surgical indication. However, the scientific community agrees in stating that patients with extensive damage of the articular cartilage of FMTPJ, or those who are affected by concurrent major deformities of the forefoot (e.g., hallux valgus), may derive limited benefits from isolated cheilectomy. In such cases, cheilectomy may lead to residual postoperative stiffness and recurrent pain; alternative interventions with a greater degree of complexity and invasiveness may then be required [10,43,44].

Successful surgery was assessed through clinical and functional outcomes using validated clinical scores, patient satisfaction questionnaires, and measuring FMTPJ mobility.

All clinical and functional outcomes improved post-operatively regardless of the approach used [22,25,26,27,28,29,30,32,33,34,36].

The postoperative VAS in studies evaluating minimally invasive techniques showed a higher mean value compared to traditional cheilectomy. Although pain is commonly thought to correlate with the severity of metatarsophalangeal joint osteoarthritis, evidence suggests that pain in hallux rigidus is influenced by a range of factors beyond joint osteoarthritis, including functional limitations, biomechanical adaptations, and overload on adjacent structures [45]. Thus, the VAS serves as a tool to reflect the multidimensional nature of pain in this condition, but it is an anatomical site and disease-related non-specific score.

The use of different outcome scores across studies makes results not fully comparable, although it was possible to perform a meta-analysis of the studies that reported the same PROMs, in this case the AOFAS score. The meta-analysis suggests a general trend towards improvement in VAS and AOFAS scores after surgery, but the substantial heterogeneity and presence of an outlier in both analyses highlight the need for caution in interpreting these results. Further research with more homogenous study designs and patient populations is needed to clarify the true impact of the intervention.

Despite a substantial improvement in the FMTPJ dorsiflexion, the mean values extracted from the present analysis still fall short of those considered physiologically normal. In healthy people, the mean dorsiflexion angle during gait is approximately 40–45° [6,46], while the mean post-operative dorsiflexion was 59.9°, though this was considered sufficient mobility.

The postoperative results in terms of FMTPJ ROM, dorsiflexion, or both, were better in patients who underwent minimally invasive cheilectomy compared to the classic technique [22,25,26,27,28,29,33,36]. This difference could be influenced by the fact that the minimally invasive technique was offered to patients who, on average, had milder forms of hallux rigidus. This consideration substantially affects the result, posing challenges in quantifying the impact of various techniques due to the diverse initial characteristics of patients.

There is growing interest in enhancing cheilectomy outcomes through various adjuvants. These include soft tissue interpositions, polyvinyl alcohol hydrogel implants, and biological supplements aimed at reducing postoperative inflammation and scar formation [47].

Recently, a randomized trial conducted by Galli et al. [25] compared isolated cheilectomy and cheilectomy associated with cryopreserved umbilical cord amniotic-membrane allograft, reporting better postoperative functional scores for the latter group. Cryopreserved umbilical cord allograft emerges as a potential adjunct to cheilectomy, exhibiting improvements in functional outcome scores compared to traditional procedures. The biological rationale reported by this study for the use of UC-AM allograft were the reduction of the inflammatory response, capability of a scaffold for epithelial cells, low risk of infection, and the “immune-privileged” status of the surgical site. However, it is essential to emphasize that this evidence is derived from a single study, and further investigations are warranted to delve deeper into this matter.

Cheilectomy had a complication rate of 11%, with most cases involving mild, transient complications or those amenable to medical and conservative therapy. The most common complication following traditional cheilectomy was postoperative pain, even though the studies discussing this technique reported better mean VAS scores for pain relief. This may be because patients undergoing traditional cheilectomy are more likely to present with severe cases, such as advanced first MTPJ osteoarthritis. Conversely, for minimally invasive cheilectomy, the most frequently reported complication was nerve injury, likely attributable to the inherent challenges of the technique, which limits adequate surgical exposure.

Revision was required in 7.4% of cases, due to onset of late complications or the progression of the arthritic process, which usually required FMTPJ arthrodesis [21,24,25,26,27,28,29,30,31,32,33,34,36]. However, no study has correlated the cases reporting worse outcomes and higher revision rates with the preoperative degree of FMTPJ osteoarthritis, which may have introduced a bias in the interpretation of the results.

The latest systematic review on this topic was published in 2010 by Roukis et al. [18]. The authors found a revision rate of 8.8% examining 706 cheilectomies with a mean follow-up of 19 months, while the current systematic review, which includes the last-ten-year literature, found a lower revision rate of 7.4% in 1179 procedures with a mean follow-up of 42.44 months.

Recent reviews have not provided data suitable for comparison. Maffulli et al. [48] included patients who underwent additional procedures alongside cheilectomy, while McNeil et al. [49] reported outcomes based on “patient satisfaction”. Neither study conducted a meta-analysis.

### Limitations

The selected studies exhibit an overall medium-low level of scientific evidence.

The relatively small number of trials selected was due to strict selection to remove potential confounders; in fact, the most common reason for exclusion was extensive resection of more than 30% of the metatarsal head, a limit set by the definition [14,16,17], or the association with other complex additional procedures.

Further limitation was a heterogeneous follow-up across the studies. Although some studies presented extended follow-up periods [21,29,34,35], some studies provide short-term follow-up that may miss late complications and underestimate the actual revision rate. Additionally, some studies measured ROM using clinical methods, while others used radiographic images.

Another limitation was the lack of protocol registration, which may introduce risks such as potential bias in the selection of studies. The authors are aware of this limitation and, given the complex nature of data interpretation in this area, it was decided to increase the flexibility with which the authors could adapt the review in response to changes during the process.

## 5. Conclusions

Based on the results, cheilectomy stands out as a common joint-sparing surgical intervention for treating hallux rigidus refractory to medical therapy. This procedure shows satisfactory functional outcomes regardless of whether the traditional or minimally invasive technique is used. However, the minimally invasive approach seems to yield better postoperative mobility. Cheilectomy also demonstrates a low re-operation rate and rare minor complications. Nevertheless, due to the low level of scientific evidence from current studies, it can be concluded that careful patient selection is critical for achieving optimal outcomes. In particular, patients with mild to moderate HR may be more likely to be satisfied after cheilectomy. In addition, surgeons need to be aware of the potential benefits and complications of both traditional and minimally invasive approaches and tailor treatment accordingly.

## Figures and Tables

**Table 1:** Coughlin and Shurnas’ classification for hallux rigidus grading.

Grade	Dorsiflexion	Radiographic Findings	Clinical Findings
0	40°–60°	Normal	No pain, only stiffness and some loss of motion
1	30°–40°	Dorsal osteophytes as main finding; minimal joint narrowing, flattening of the metatarsal head, and/or periarticular sclerosis	Mild and/or intermittent pain and stiffness at maximal dorsiflexion and/or plantar flexion of the joint
2	10°–30°	Periarticular osteophytes with mild to moderate joint narrowing, flattening of the metatarsal head, and/or periarticular sclerosis	Moderate to severe pain and stiffness with a more pronounced frequency; pain evoked near end range of motion of the joint
3	Less than or equal to 10°	Same as grade 2 but with the addition of cystic changes subchondrally and likely sesamoid irregularities	Nearly constant pain and stiffness with the pain being elicited with end range of motion, but not at midrange
4	Same as grade 3	Same as grade 3	Same as grade 3 but with pain present at midrange of passive motion of the joint

**Table 2:** Quality assessment using the Newcastle–Ottawa scale for included studies.

Authors and Years	Representativeness of Cases	Selection of Controls	Ascertainment of Exposure	Demonstration That Outcome of Interest Was Not Present at Start of Study	Comparability	Assessment of Outcome	Follow-Up Long Enough	Adequacy of Follow-Up	Total
Brandao, B. et al.—2020 [21]	-	-	★	★	★	★	★	★	6★
Cetinkaya, E. et al.—2016 [22]	-	-	★	★	-	★	★	★	5★
Cöster, M.E. et al.—2021 [23]	-	-	★	★	-	★	-	★	4★
Cullen, B. et al.—2017 [24]	-	-	★	★	★	★	★	★	6★
Galli, S.H. et al.—2021 [25]	-	-	★	★	★	★	★	★	6★
Gauthier, C. et al.—2024 [26]	-	-	★	★	-	★	★	★	5★
Glenn, R. L. et al.—2021 [27]	-	-	★	★	-	★	-	★	4★
Hickey, B.A. et al.—2020 [28]	-	-	★	★	-	★	★	★	5★
Hoskins, T. et al.—2023 [29]	-	-	★	★	★	★	★	★	6★
Lee, J.Y. et al.—2021 [30]	-	-	★	★	★	★	★	★	6★
Jones, M.D. et al.—2018 [31]	-	-	★	★	-	★	-	★	4★
Kim, J. et al.—2023 [32]	-	-	★	★	★	★	-	★	5★
Pastides, P.S. et al.—2014 [33]	-	-	★	★	-	★	★	★	5★
Sidon, E. et al.—2019 [34]	-	-	★	★	-	★	★	★	5★
Stevens, J. et al.—2020 [35]	-	-	★	★	★	★	★	★	6★
Teoh, K.H. et al.—2019 [36]	-	-	★	★	-	★	★	★	5★

Note: The total quality score ranged from 0 to 9, with studies scoring ≥5 points being considered at low risk of bias, while those scoring <5 points were classified as high risk of bias. A study can be given a maximum of one star for each numbered item within the Selection and Outcome categories. A maximum of two stars can be given for Comparability.

**Table 3:** Studies design and patients’ characteristics.

Authors—Years	Study Design and LOE	Number of Patients (Halluces)	Gender F:M	Age at Surgery (Years)	Type of Surgical Procedure	Classification and Grades	FU (Months)
Brandao, B. et al.—2020 [21]	Prospective comparative study; LOE II	23 (23)	19:4	58 ± 10.25 (38–79)	Traditional cheilectomy	Hattrup and Johnson Grade I = 5 Grade II = 10 Grade III = 8	69 ± 10.9 (50–90)
Cetinkaya, E. et al.—2016 [22]	Retrospective case series; LOE IV	21 (22)	14:7	59.2 ± 3.75 (52–67)	Traditional cheilectomy	Coughlin-Shurnas Grade III = 22	24.8 ± 12.75 (12–63)
Cöster, M.E. et al.—2021 [23]	Retrospective case series; LOE IV	181 (181)	112:69	58 ± 11	Traditional cheilectomy	Coughlin-Shurnas Grade II = 70 Grade III = 111	12
Cullen, B. et al.—2017 [24]	Retrospective comparative study; LOE III	330 (340)	206:124	-	Traditional cheilectomy	Coughlin-Shurnas Grade II = 340	39.2
Galli, S.H. et al.—2021 [25]	Prospective comparative study; LOE II	51 (58)	36:15	51.7 ± 9.1 (34.7–71.1)	Cheilectomy vs. cheilectomy + (UC-AM) allograft	-	44.9
Gauthier, C. et al.—2024 [26]	Retrospective case series; LOE IV	31 (31)	21:10	54.2 ± 11.6 (26.2–80.8)	Minimally invasive cheilectomy in combination with FMTPJ arthroscopy	-	16.5 ± 4.5 (12.0–26.2)
Glenn, R.L. et al.—2021 [27]	Retrospective case series; LOE IV	20 (20)	14:6	52 ± 7.25 (40–69)	Minimally invasive cheilectomy in combination with FMTPJ arthroscopy	-	16.5 ± 7.5 (3–33)
Hickey, B.A. et al.—2020 [28]	Retrospective case series; LOE IV	36 (36)	26:10	50.0 ± 10.62 (24.5–67.0)	Minimally invasive cheilectomy in combination with FMTPJ arthroscopy	Coughlin-Shurnas Grade I = 5 Grade II = 27 Grade III = 1 Not reported = 3 Outerbridge classification: 3.39 ± 1.78, range 0–8	56.3 ± 15.9 (24–87.6)
Hoskins, T. et al.—2023 [29]	Retrospective comparative study; LOE III	30 (31)	20:10	57 ± 8 (43–75)	Traditional cheilectomy	-	67 ± 16.75 (39–106)
Lee, J.Y. et al.—2021 [30]	Retrospective comparative study; LOE III	17 (17)	12:5	54.8 ± 7.82 (41.5–72.8)	Traditional cheilectomy	Hattrup and Johnson Grade I = 2 Grade II = 6 Grade III = 9	24
Jones, M.D. et al.—2018 [31]	Retrospective comparative study; LOE III	26 (26)	-	-	Traditional cheilectomy	Drago, Oloff and Jacobs scoring system Grade I = 3 Grade II = 16 Grade III = 7	-
Kim, J. et al.—2023 [32]	Retrospective comparative study; LOE III	62 (62)	42:20	51.18	Traditional cheilectomy	Coughlin-Shurnas Grade I = 4 Grade II = 22 Grade III = 28 Grade IV = 6 Not reported = 2	20.5
Pastides, P.S. et al.—2014 [33]	Retrospective case series; LOE IV	41 (54)	35:6	43 ± 11.25 (16–61)	Minimally invasive cheilectomy without FMTPJ arthroscopy	Coughlin-Shurnas Grade I = 9 Grade II = 19 Grade III = 26	17 ± 6 (6–30)
Sidon, E. et al.—2019 [34]	Retrospective case series; LOE IV	165 (169)	110:55	54.2 ± 14.5 (18–76)	Traditional cheilectomy	Hattrup and Johnson Grade I = 30 Grade II = 118 Grade III = 21	79.2 ± 17.7 (60–130.8)
Stevens, J. et al.—2020 [35]	Retrospective comparative study; LOE III	10 (11)	4:6	51.4 ± 7.0 (39–62)	Traditional cheilectomy	-	271.1 ± 27 (228–336)
Teoh, K.H. et al.—2019 [36]	Retrospective case series; LOE IV	89 (98)	64:25	54 ± 10.5 (29–71)	Minimally invasive cheilectomy without FMTPJ arthroscopy	Coughlin-Shurnas Grade I = 33 Grade II = 54 Grade III = 11	50 ± 18 (12–84)
Mean				54.14			42.44
Tot		1133 (1179)	735F:372M		Traditional cheilectomy = 11 Minimally invasive cheilectomy = 5	Hattrup and Johnson Grade I = 37 Grade II = 134 Grade III = 38 Grade IV = 0 Coughlin-Shurnas Grade I = 51 Grade II = 532 Grade III = 199 Grade IV = 6	

Abbreviations: LOE: Level of evidence; FU: Follow-up; F: female; M: male.

**Table 4:** Clinical outcomes and Range of Motion.

Authors and Years	N° Patients (Halluces)	VAS Pre	VAS Post	AOFAS Pre	AOFAS Post	Other Clinical Scores	Range of Motion	Dorsiflexion	Plantarflexion
Brandao, B. et al.—2020 [21]	23 (23)	-	-	-	-	MOXFQ Index: 14 FAAM Sports subscale 82.7%	-	-	-
Cetinkaya, E. et al.—2016 [22]	21 (22)	8.9 ± 1 (6.0–10.0)	2.9 ± 1.75 (0–7.0)	53 ± 9.5 (29–67)	78 ± 8.75 (57–92)	-	Pre-op 13° ± 4.75 (5°–24°) Post-op 41° ± 1.75 (24°–31°)	-	-
Cöster, M.E. et al.—2021 [23]	181 (181)	-	-	-	-	SEFAS: pre-op 26 post-op 36 EQ-5D index: pre-op 0.61 Post-op 0.77	-	-	-
Cullen, B. et al.—2017 [24]	330 (340)	-	-	-	-	-	-	-	-
Galli, S.H. et al.—2021—Cheilectomy [25] *	25 (27)	4.6 ± 1.65 (0.7–7.3)	0.66 ± 0.62 (0–2.5)	69 ± 2 (64–72)	81.5 ± 2.1 (76.5–85)	Foot function index: mean 26.3	-	-	-
Galli, S.H. et al.—2021— Cheilectomy + UC-AM [25] *	26 (31)	5.2 ± 1.82 (1–8.3)	0.86 ± 0.85 (0–3.4)	65 ± 1.25 (62–67)	90 ± 3.25 (82–95)	-	-	-	-
Gauthier, C. et al.—2024 [26]	31 (31)	6.5 ± 2.7	2.1 ± 1.9	-	-	EQ-5D index: pre. 0.64 ± 0.2 post. 0.76 ± 0.21 MOXFQ Index: pre. 54.7 ± 18.9 post. 22.4 ± 23.5	Pre-op 61.3° Post-op 108.2°	Pre-op 50 ± 20.8 Post-op 89.6 ± 1.3	Pre-op 11.3 ± 9.6 Post-op 18.6 ± 7.8
Glenn, R.L. et al.—2021 [27]	20 (20)	7.05 ± 1.25 (5–10)	0.75	-	-	-	Pre-op 47° Post-op 67°	Pre-op 32° ± 12.5 (10°–60°) Post-op 48°	Pre-op 15° ± 7.5 (0°–30°) Post-op 19°
Hickey, B.A. et al.—2020 [28]	36 (36)	-	2.7	-	-	-	-	Pre-op 31.86° ± 9.71 (10°–50°) Post-op 72.71° ± 10.80 (45°–90°)	-
Hoskins, T. et al.—2023 [29]	30 (31)	-	-	49.6 ± 11.7	85.3 ± 8.9	FAOS: pre. 55.5 ± 14.0 post 88.4 ± 8.6	Pre-op 40.9° Post-op 57.1°	Pre-op 24° ± 10.2 Post-op 38° ± 7.8	Pre-op 16.9° ± 5.4 Post-op 19° ± 5.2
Lee, J.Y. et al.—2021 [30]	17 (17)	6.1 ± 2.2	1.7 ± 2.7	46.9 ± 14.2	78.8 ± 21.0	SF-36 subscales: PCS: pre. 42.5 ± 10.7 post. 48.3 ± 11.2 MCS: pre. 56.2 ± 7.8 post. 52.2 ± 11.7	-	-	-
Jones, M.D. et al.—2018 [31]	26 (26)	-	-	-	-	Maryland Foot Score: 83.1 *	-	-	-
Kim, J. et al.—2023 [32]	62 (62)	-	-	-	-	PROMIS: pre- and postoperative [37]	-	-	-
Pastides, P.S. et al.—2014 [33]	35 (47)	-	-	71.1	87.1	-	Pre-op 39° Post-op 48°	Pre-op 32° ± 7.25 (15–44) Post-op 39° ± 7 (28–56)	Pre-op 7° ± 2.5 (0–10) Post-op 9° ± 2.5 (5–15)
Sidon, E. et al.—2019 [34]	165 (169)	6.4 ± 2.5	1.1 ± 2.3	-	-	-	-	-	-
Stevens, J. et al.—2020 [35]	10 (11)	2	1.81 ± 2.28 (0–7.1)	79.8	77.1 ± 27.2 (24–100)	Forgotten joint score-12: 71.8 ± 30.7 (25–100) * MOXFQ Index: 26 ± 24.9 (0–70.3)	43.1 ± 18.7	Post-op 24.6° ± 19.0 (10°–55°)	Post-op 18.5°
Teoh, K.H. et al.—2019 [36]	89 (98)	8.0 ± 1 (6–10)	3 ± 2.5 (0–10)	-	-	-	-	Pre-op 11.3° ± 7.5 (0°–30°) Post-op 69.1° ± 10 (50°–90°)	-
Mean	1127 (1172)	Pre-op: 6.60	Post-op: 1.76	Pre-op: 62.35	Post-op: 84.04	-	Pre-op: 38.43° Post-op: 62.32°	Pre-op: 25.44° Post-op: 59.88°	Pre-op: 11.65° Post-op: 15.51°

Note: Studies that reported clinical outcomes separately to compare different techniques were treated as distinct studies (marked with *).

**Table 5:** Comparison of VAS and ROM between techniques.

		Traditional Cheilectomy							
Author/Year	Halluces	VAS Pre-Op	VAS Post-Op	Mean ROM Pre-Op	Mean ROM Post-Op	Dorsiflexion Pre-Op	Dorsiflexion Post-Op	Plantarflexion Pre-Op	Plantarflexion Post-Op
Cetinkaya et al.—2016 [22]	22	8.9 ± 1	2.9 ± 1.75	13° ± 4.75	41° ± 1.75	-	-	-	-
Galli, S.H. et al.—2021 [25]	27	4.6 ± 1.65	0.66 ± 0.62	-	-	-	-	-	-
Hoskins, T. et al.—2023 [29]	31	-	-	40.9°	57.1°	24 ± 10.2°	38 ± 7.8°	16.9 ± 5.4°	19 ± 5.2°
Lee, J.Y. et al.—2021 [30]	17	6.1 ± 2.2	1.7 ± 2.7	-	-	-	-	-	-
Sidon, E. et al.—2019 [34]	169	6.4 ± 2.5	1.1 ± 2.3	-	-	-	-	-	-
Stevens, J. et al.—2020 [35]	11	2	1.8 ± 2.28	-	43.1 ± 18.7°	-	24.6 ± 19.0°	-	18.5°
Tot	277								
Mean		6.20	1.28	29.31°	49.15°	24°	34.49°	16.9°	18.86°
		**Mini-Invasive Cheilectomy**							
**Author/Year**	**Halluces**	**VAS Pre-Op**	**VAS Post-Op**	**Mean** **ROM Pre-Op**	**Mean** **ROM Post-Op**	**Dorsiflexion Pre-Op**	**Dorsiflexion Post-Op**	**Plantarflexion Pre-Op**	**Plantarflexion Post-Op**
Gauthier C. et al.—2024 [26]	31	6.5 ± 2.7	2.1 ± 1.9	61.3°	108.2°	50 ± 20.8°	89.6 ± 1.3°	11.3 ± 9.6°	18.6 ± 7.8°
Glenn R.L. et al.—2021 [27]	20	7.05 ± 1.25	0.75	47°	67°	32 ± 12.5°	48°	15 ± 7.5°	19°
Hickey B.A. et al.—2020 [28]	36	-	2.7	-	-	31.86 ± 9.71°	72.71 ± 10.8°	-	-
Pastides, P.S. et al.—2014 [33]	47	-	-	39°	48°	32 ± 7.25°	39 ± 7°	7 ± 2.5°	9 ± 2.5°
Teoh K.H. et al.—2019 [36]	98	8 ± 1	3 ± 2.5	-	-	11.3 ± 7.5°	69.1 ± 10°	-	-
Tot	232								
Mean		7.28	2.55	47.68°	70.92°	25.64°	64.48°	9.99°	14.07°

**Table 6:** Comparison of complications between techniques.

Traditional Cheilectomy						
Author/Year	Halluces	Infection	EHL Lesion	Nerve Injury	Postoperative Pain	Delayed Wound Healing
Brandao, B. et al.—2020 [21]	23	-	-	-	-	-
Cetinkaya et al.—2016 [22]	22	-	-	3	-	-
Galli, S.H. et al.—2021 [25]	27	-	-	-	2	2
Hoskins, T. et al.—2023 [29]	31	-	-	-	6	-
Lee, J.Y. et al.—2021 [30]	17	-	-	-	-	-
Kim, J. et al.—2023 [32]	62	1	-	-	5	-
Sidon, E. et al.—2019 [34]	169	-	-	-	22	-
Tot	351	1 (0.28%)	0	3 (0.85%)	35 (9.97%)	2 (0.56%)
Mini-invasive Cheilectomy						
Gauthier, C. et al.—2024 [26]	31	-	1	-	1	-
Glenn, R.L. et al.—2021 [27]	20	-	-	-	-	-
Hickey, B.A. et al.—2020 [28]	36	-	1	3	4	-
Teoh, K.H. et al.—2019 [36]	98	2	-	4	-	2
Tot	185	2 (1.08%)	2 (1.08%)	7 (3.78%)	5 (2.70%)	2 (1.08%)
All approaches						
	536	3 (0.55%)	2 (0.37%)	10 (1.86%)	40 (7.46%)	4 (0.74%)

## Data Availability

Not applicable.
